# Frequency Nesting Interactions in the Subthalamic Nucleus Correlate With the Step Phases for Parkinson’s Disease

**DOI:** 10.3389/fphys.2022.890753

**Published:** 2022-04-29

**Authors:** Luyao Jin, Wenbin Shi, Chuting Zhang, Chien-Hung Yeh

**Affiliations:** ^1^ School of Information and Electronics, Beijing Institute of Technology, Beijing, China; ^2^ Nuffield Department of Clinical Neurosciences, University of Oxford, Oxford, United Kingdom

**Keywords:** auditory cue, Parkinson’s disease, phase-amplitude coupling, gait phase, masking PAC

## Abstract

Gait disturbance in Parkinson’s disease (PD) can be ameliorated by sound stimulation. Given that excessive *β* synchronization in basal ganglia is linked to motor impairment in PD, whether the frequency nesting interactions are associated with the gait problem is far from clear. To this end, the masking phase-amplitude coupling (PAC) method was proposed to overcome the trade-off between intrinsic nonlinearity/non-stationarity and demand for predetermined frequencies, normally extracted by the filter. In this study, we analyzed LFPs recorded from 13 patients (one female) with PD during stepping with bilateral deep brain electrodes implanted in the subthalamic nucleus (STN). We found that not only high-frequency oscillation (100–300 Hz) was modulated by *β* (13–30 Hz) but also *β* and *γ* amplitude were modulated by their low-frequency components in *δ*/*θ*/*α* and *δ*/*θ*/*α*/*β* bands. These PAC values were suppressed by sound stimulation, along with an improvement in gait. We also showed that gait-related high*-β* (*Hβ*) modulation in the STN was sensitive to auditory cues, and *Hβ* gait-phase modulation increased with a metronome. Meanwhile, phase-locking values (PLVs) across all frequencies were significantly suppressed around contralateral heel strikes, manifesting the contralateral step as a critical gait phase in gait initiation for PD. Only the PLVs around contralateral steps were sensitive to auditory cues. Our results support masking PAC as an effective method in exploring frequency nesting interactions in LFPs and reveal the linkages between sound stimulation and couplings related to gait phases in the STN. These findings raise the possibility that nesting interactions in the STN work as feasible biomarkers in alleviating gait disorders.

## Introduction

Gait disturbance, independent of other cardinal symptoms (Schaafsma et al., 2003), is refractory to medication and deep brain stimulation (DBS) (Smith et al., 2012; Pötter-Nerger and Volkmann, 2013; Zhou et al., 2019). Excessive *β*-band power (13–30 Hz) in basal ganglia (BG) is linked to motor impairment in PD, wherein the suppression correlates with clinical improvement (Kühn et al., 2008; Little and Brown, 2014). In contrast, *γ* (60–90 Hz) synchrony acts as a prokinetic role and facilitates inter-regional communication (Fries et al., 2007; Buzsáki and Wang, 2012). The cross-frequency interactions of neuronal synchrony are associated with functional activities (Jensen and Colgin, 2007; Canolty and Knight, 2010). Studies showed abnormal neuronal synchronization in LFP/EEG is associated with gait problems (Singh et al., 2013; Shine et al., 2014). However, cross-frequency interactions of BG activities in gait remain unclear. Untangling the underneath cross-frequency interactions during stepping could support advances in adaptive therapies.

Phase-amplitude coupling (PAC) is a measure that evaluates cross-frequency interactions between low-frequency phase and high-frequency amplitude. The PAC has been observed in the primary motor cortex (M1) and subthalamic nucleus (STN) for PD patients while resting. In STN for PD, pathophysiological coupling occurs between the high-*β* (*Hβ*, 20–35 Hz) phase and high-frequency oscillation (HFO, 250–400 Hz) (Wang et al., 2016). The broadband-*γ* (50–200 Hz) amplitude is modulated by the *β* phase in M1 for PD (de Hemptinne et al., 2013; de Hemptinne et al., 2015). Pathophysiological PAC mentioned earlier could be suppressed by DBS (Yang et al., 2014; de Hemptinne et al., 2013, de Hemptinne et al., 2015. However, the fact whether PAC in the STN shifts during gait for PD remains unclear. Our past work showed *β* modulation in the STN is time-locked to the gait phase in PD (Fischer et al., 2018), and the gait phase modulates low-*γ* (25–40 Hz) oscillations in the motor cortex (Wagner et al., 2012; Seeber et al., 2014) for healthy humans. We hypothesized that antagonism between *γ* and *β* modulation in the STN is reflected by the PAC time-locked to the step phase. We also investigated whether the PAC across other frequency bands varied with the step phase. We aimed to explore frequency nesting interactions time-locked to the movement of lower limbs.

Fourier decomposition is straightforward in constituting orthogonal functions for PAC, but its linear/stationary nature distorts irregularity of intrinsic rhythmicity, resulting in harmonic distortion and spurious coupling (Yeh et al., 2016). Empirical mode decomposition (EMD) is known to adaptively decompose an irregular oscillation into intrinsic mode functions (IMFs) (Huang et al., 1998). However, the dyadic-filter-bank property of EMD (Flandrin et al., 2004) limits its direct use to estimate the PAC between predetermined frequencies. Recently, the masking EMD, which had been justified as an effective method in exploring PAC (Yeh and Shi, 2018), was proposed to deal with the trade-off between the nonlinearity of intrinsic rhythmicity and the demand for narrow bands. Given that cross-couplings prevail in neural activities, finer divisions in frequency bands infer more explicit computation in nesting neural interactions.

Auditory cues, by acting as an internal clock to regulate rhythm formation processes, improve gait for PD patients (Hausdorff et al., 2007; Arias and Cudeiro, 2008; Nombela et al., 2013). Thus, our last hypothesis is that auditory cues suppress pathophysiological PAC in the STN, thus liberating step-related neural modulation (e.g., *γ* synchrony) for parkinsonism.

## Materials and Methods

### Patients and Experiments

A total of 13 patients [age 61 ± 4 years (mean ± SD), mean disease duration 12 ± 4 years, 1 female] with PD who had undergone bilateral implantation of DBS electrodes in the STN were included in this work (Supplementary Table S1). Of note, the electrodes were used to collect signals, instead of acting DBS. The experiment was approved by the local ethics committee with all patients giving their informed consent. The experiment was performed 3–7 days after the surgery without withdrawal of dopaminergic medication. Either quadripolar (Medtronic 3,389, n = 8) or octopolar DBS electrodes (Boston Scientific DB-2201 Vercise and DB-2202 Vercise directional correspond to n = 3 and 2, respectively) were used for the electrode implantation.

Patients were instructed to sit in a chair and step in place rhythmically with the upper limb put on the lap. They were asked to step alternatively by synchronizing their steps to the instructional video with each side lasting for one second. The timing of each footstep was annotated by a dual-plate pressure sensor placed on the floor. Nine patients were provided with a metronome sound at the time of each heel strike displayed in the instructional video during the mid-period of stepping [S3-S11 in Supplementary Table S1], while four patients without cues [S1, S2, S12, and S13] were considered as the before-sound condition. The electrode pairs with impact-related broadband artifacts and sporadic steps with strong movement artifacts (e.g., cable movement) were discarded, following a visual inspection. STN-LFPs were recorded in monopolar configuration with a TMSi Porti amplifier (TMS International). Common averaged LFPs were sampled at 2048 Hz, then re-referenced to a spatially focal bipolar signal (i.e., the bipolar contact pair or the bipolar channel), and downsampled to 1,000 Hz for all the analyses. All data are publicly accessible on the data-sharing platform of BNDU-MRC (https://data.mrc.ox.ac.uk/data-set/).

### Data Preprocessing and Time–Frequency Analysis

Raw LFPs in bipolar configurations were notch filtered at 50 Hz to suppress power line noise, followed by a sixth-order high-pass Butterworth filter with a cut-off frequency set at 1 Hz to remove the low-frequency artifacts. The processed LFPs were divided into epochs according to the timing of heel strikes, of which each 2-s epoch spans from 0.5 s before a contralateral heel strike to 0.5 s after the next ipsilateral heel strike. These epochs were linked in chronological order for further analysis.

The time–frequency analysis in this work was conducted with continuous Morlet wavelet transform with the wavelets set to span six cycles. The resulting time–frequency decomposition was smoothed with a sliding window set as 0.2 s. Relative power was calculated per subject per channel by normalizing the absolute power referenced to its average across time. The step-timing variability was also computed as a sign of gait performance, which was defined as the median absolute deviation of the nearest difference between real and instructed steps (Fischer et al., 2018). Of note, lower step-timing variability implies superior gait performance.

### Masking Phase-Amplitude Coupling

**FIGURE 1:**
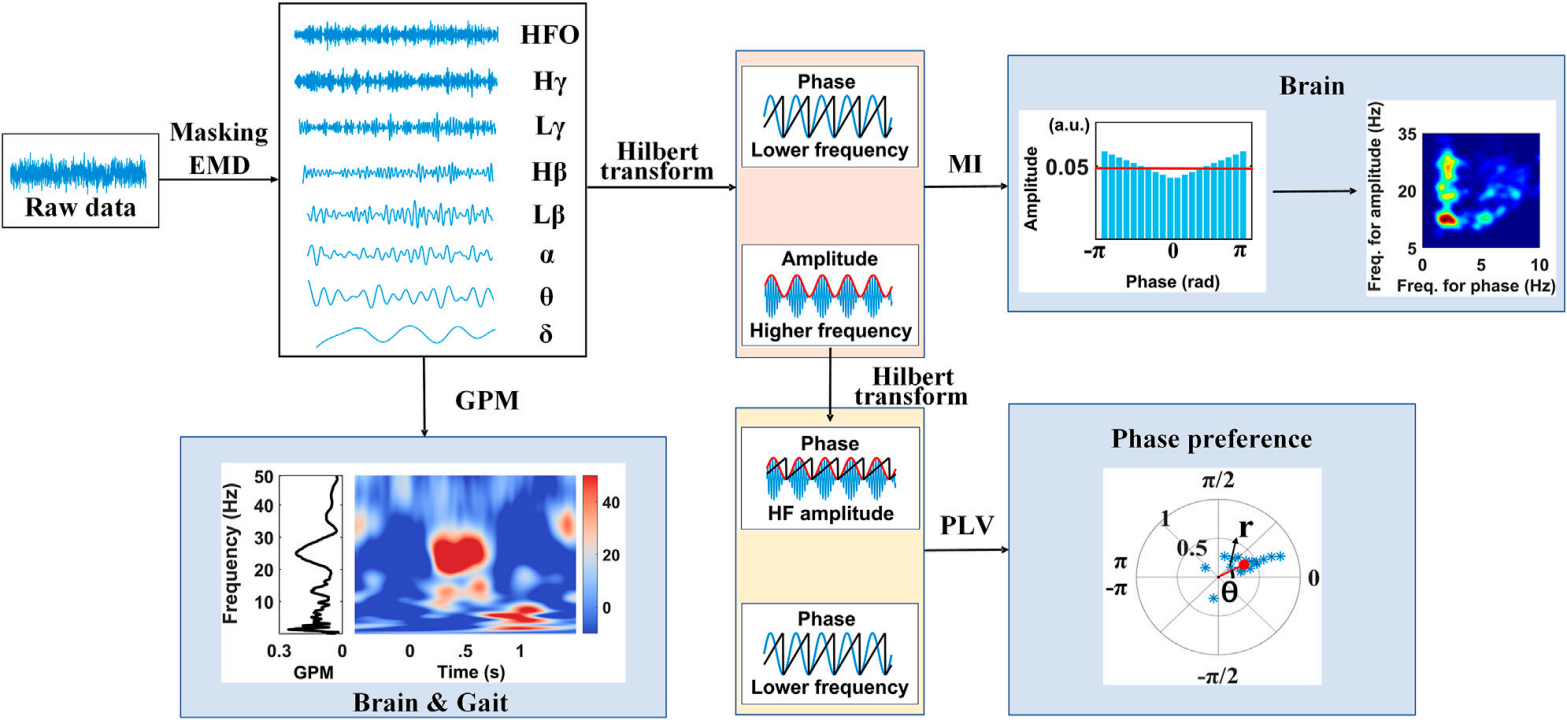
Graphical overview of the study.

The procedure for estimating masking phase-amplitude coupling (MPAC) is illustrated in the upper panel of Figure 1 and begins with finding intrinsic activities in different frequencies. EMD, a method to decompose an irregular oscillation into an orthogonal set of IMFs, is an ideal approach to reserving the nonlinearity of an intrinsic activity (Huang et al., 1998). However, as EMD behaves like a dyadic filter bank, i.e., the harmonic sources are allotted into individual IMFs, respectively, the distribution of frequency for each IMF may not fit well with the classical frequency bands of interest in brain activities (e.g., *β*-band, *γ*-band, etc.). Meanwhile, MPAC is known for its ability of extracting the specific frequency bands, as well as simultaneously minimizing the mode mixing, the mode splitting effect, and the residual effects (Wang et al., 2018; Yeh and Shi, 2018). To this end, assisted sinusoid signals *s*(*t*) were designed, i.e., $s_{n}\left(t\right)=a_{d}\cos\left(2\pi f_{a}t+\varphi_{n}\right)=a_{d}\cos\left(2\pi f_{a}t+2\pi n/N\right)$ , wherein *φ*_*n*_ stands for the phase and the amplitude *a*_*d*_ equals the standard deviation of the original signal. $f_{a}=p\cdot f_{d}\cdot 2^{k}$ , where *f*_*d*_ stands for the desired frequency, *p* is an empirical parameter, *k* represents an integer, and of note, starting from the highest, *f*_*a*_ follows the Nyquist law. Next, EMD was applied to decompose an individual constructed signal into IMFs, of which the first IMF comprises the highest frequency component along with the assisted sinusoid signals (Deering and Kaiser, 2005). The masking signals were designed with the phase-step *φ*_*n*_ ranging equally between 0 and 2*π* to guarantee a complete cancellation of the added sinusoid, i.e., $y_{n}\left(t\right)=x\left(t\right)+s_{n}\left(t\right)=\overset{M}{\underset{i=1}{\sum }}c_{i,n}\left(t\right)+r_{n}$ , so that the highest frequency component of *x*(*t*) was calculated by $C=\overset{N}{\underset{n=1}{\sum }}c_{1,n}\left(t\right)/N$ . Repeating the abovementioned procedure to the remains (i.e., *x*(*t*)-*C*), k=k−1 , the second highest frequency component was obtained until the desired frequency was obtained. In this work, we targeted the classical frequencies of interest in LFPs including *δ*, *θ*, *α*, low*-β* (*Lβ*), *Hβ*, *Lγ*, high-*γ* (*Hγ*), and HFO, respectively.

Next, the modulation index (MI) was used to describe the PAC between the decompositions of interests, which were selected from IMFs (Tort et al., 2010). The algorithm is summarized as follows: the analytical representations of decompositions were used to form the instantaneous phase and amplitude series, of which the high-frequency amplitude at each phase bin (*N* = 20 bins in this work) of the low-frequency component was averaged to form distribution *P*. Then, the Kullback–Leibler divergence *D*_*KL*_ from a uniform amplitude distribution *U* is defined as MI, i.e., $MI=D_{KL}\left(P,U\right)/logN$ , where $D_{KL}\left(P,U\right)=logN+\overset{N}{\underset{1}{\sum }}P\left(i\right)logP\left(i\right)$ . The MI was validated with the bootstrap strategy. Meanwhile, a comodulogram was used to visualize the PACs. Briefly, MIs of all frequency pairs were expressed to a bidimensional frequency plane; according to the cycle-by-cycle frequency at each time point, comodulograms were obtained by averaging the corresponding MI values in the predetermined regions of bidimensional frequency planes (Tort et al., 2008; Pittman-Polletta et al., 2014). In this work, the phase frequency spans from 0.1 to 10 Hz (frequency resolution = 0.1 Hz), whereas the amplitude frequency ranges from 5 to 35 Hz (frequency resolution = 0.25 Hz).

The phase-locking value (PLV), on the other side, emphasizes the phase synchrony between two oscillations (Cohen, 2008; Penny et al., 2008) and is defined as the value of the mean phase difference between the two oscillations expressed as a complex form. Here, we calculated the PLV between the high-frequency amplitude *φ*_*HFA*_ and low-frequency oscillations *φ*_*LFO*_, i.e., $PLV=\frac{1}{n}\left|\overset{n}{\underset{i=1}{\sum }}e^{i\left(\varphi_{LFO}-\varphi_{HFA}\right)}\right|$ .

### Gait Phase Modulation

To identify if the LFP rhythmic envelope is phase-locked to the gait cycle, the gait phase modulation (GPM), a revised version of MI, was introduced. To begin with, Morlet wavelets spanned at six cycles were applied to obtain temporal oscillations of STN-LFPs in different frequencies from 1 to 90 Hz with 0.5 Hz in steps. Relative amplitude changes *a* (*f*, *n*) for each time point were calculated by normalizing the absolute value relative to its average across a gait cycle at each frequency bin. The magnitude of GPM expresses the correlation between the LFP frequency component and a gait step–based sinusoid, which has a maximal value of 1 on the condition of the LFP rhythmic envelope modulating sinusoidally with the gait phase (Seeber et al., 2014, 2015). The phase lag of the LFP rhythmic envelope and gait phase is also accessible using GPM. The GPM is defined as $GPM\left(f\right)=\left(\sqrt{2}/\sigma N\right)\cdot \overset{N-1}{\underset{n=0}{\sum }}a\left(f,n\right)\cdot e^{-i2\pi n/N}$ , where *a* (*f*, *n*) represents the relative amplitude change at a frequency *f* and the sample point *n*; *N* stands for the number of sample points within a gait cycle, while *σ* denotes the standard deviation of *a* (*f*).

### Statistical Analyses

The significance tests aim to examine the performances of PAC and PLVs in the STN, as well as GPMs between STN-LFP rhythmicity and gait cycle, which is sensitive to gait steps and auditory stimulation, and thus may serve as critical features in identifying the different states of gaits for patients with PD. To this end, a general linear mixed model (GLMM) was used to assess the impacts of the fixed effects, including the sound conditions (three conditions include before-sound, sound-on, and after-sound conditions) as well as the step phases (two or four phases according to the timing of heel strikes), to the shifts of cross-frequency neural interactions within the STN *per se* or to the gait. Subjects and hemispheres are set as random factors to avoid their interferences. The Shapiro–Wilk test was applied to test the assumption of normality. A two-tailed *p*-value < 0.05 was considered statistically significant for all hypothesis testing (*α* = 0.05). We implemented Tukey’s honest significance test (Tukey’s HSD) to test all possible pairwise differences of means as the correction for multiple comparisons. Results are reported as mean ± standard error. All data were analyzed in MATLAB (MathWorks, Natick, MA). All statistical tests were performed using JMP (Business Unit of SAS).

## Results

In this work, LFPs were recorded from the STN for patients with PD who had undergone bilateral implantation of DBS electrodes. The experiment was performed during stepping either before, on, or after a set of auditory cues. As shown in Figure 1, raw LFPs were decomposed by the masking EMD and produced components in multiple frequency bands including *δ* (1–4 Hz), *θ* (4–8 Hz), *α* (8–13 Hz), *Lβ* (13–20 Hz), *Hβ* (20–30 Hz), *Lγ* (30–60 Hz), *Hγ* (60–90 Hz), and HFO (100–300 Hz), which are representative of pathophysiological characteristics, along with their instantaneous phase and amplitude time series calculated using the Hilbert transform. Then, either the PAC and the phase synchronize across multiple frequencies within the STN or the gait phase–locked amplitude modulations in the STN were carefully explored.

### The Sound Stimulation Effect on *β*-HFO PAC During Stepping

**FIGURE 2:**
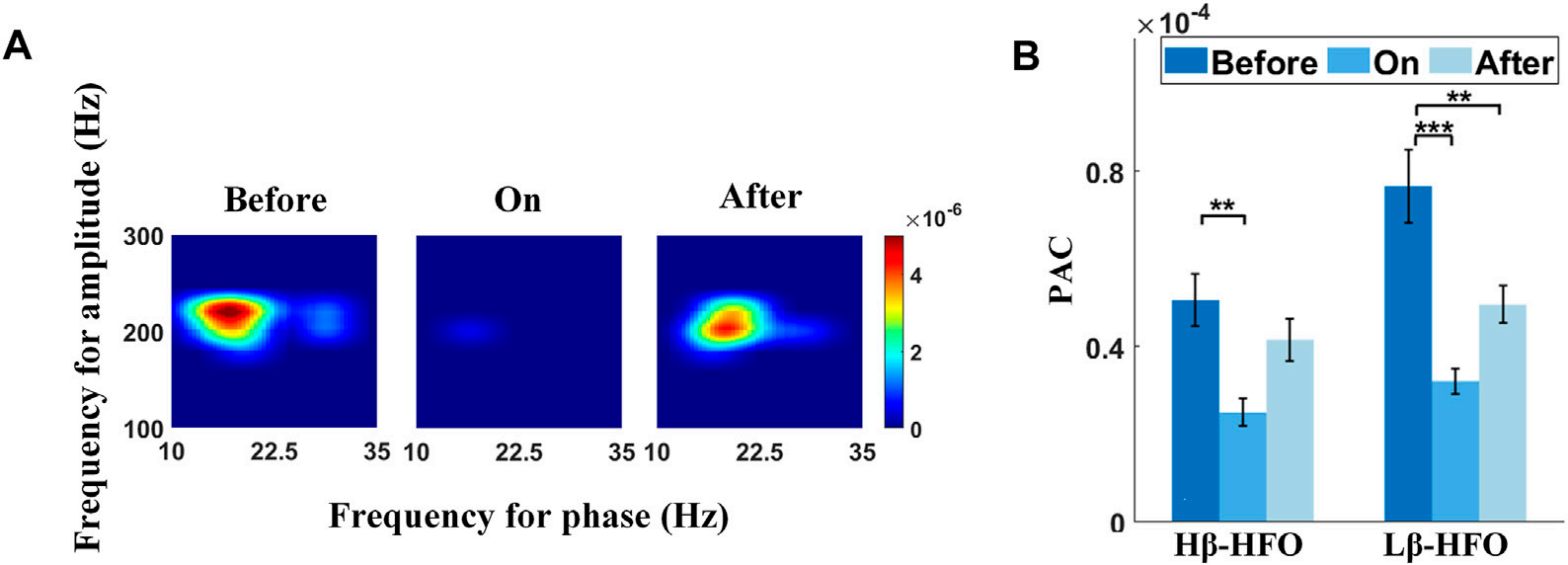
Effect of sound stimulation on *Lβ*/*Hβ-*HFO PAC. **(A)** Typical example shows the emergence of excessive *Lβ*/*Hβ-*HFO PAC in the STN for a patient with PD (left panel), while sound stimulation suppresses the PAC (middle panel), followed by a rebound after removing auditory cues (right panel). **(B)** Comparisons of *Hβ*-HFO PAC and *Lβ*-HFO PAC between three different sound conditions. Both PAC values show decreased coupling strength with sound stimulation, followed by a rebound after withdrawal of auditory cues, especially for *Lβ*-HFO PAC. Of note, *, **, and *** correspond to *p* < 0.05, 0.01, and 0.001, respectively.

In this study, excessive PAC between the *Lβ*/*Hβ* phase (10–30 Hz) and HFO amplitude (100–300 Hz) were detected as expected (de Hemptinne et al., 2013; Wang et al., 2016). The effect of sound stimulation on *Lβ*/*Hβ-*HFO PAC in the STN from a representative channel is shown in Figure 2. The left, the center, and the right panels of Figure 2A correspond to the comodulograms before, during, and after sound stimulation, respectively, of which each of the lower frequency ranges from 10 to 35 Hz in steps of 0.5 Hz, while the higher frequency (100–300 Hz) increases by 2 Hz. Strong PAC between the 10–22 Hz phase and 180–240 Hz amplitude, along with a relatively weaker coupling strength between the 26–30 Hz phase and 190–240 Hz amplitude were revealed before auditory cues. Both PACs were suppressed with auditory cues, followed by a rebound after removing cues. The corresponding step-timing variabilities are 0.29, 0.09, and 0.18 s, respectively (from left to right panels in Figure 2A).

Figure 2B shows the performances of the significance tests for *Lβ*/*Hβ*-HFO PAC with sound conditions (i.e., before-sound, sound-on, and after-sound) as the fixed effect. Both *Lβ*-HFO PAC (F = 12.42, *p* < 0.0001) and *Hβ*-HFO PAC (F = 6.42, *p* = 0.0020) show significant differences among various sound conditions (Supplementary Table S2), wherein both PACs show significant lower coupling strength during sound stimulation than that before cues (*p* < 0.001 and *p* < 0.01 for *Lβ*-HFO PAC and *Hβ*-HFO PAC, respectively), while only *Lβ*-HFO PAC presents significant lower PAC after removing auditory cues than that before cues (*p* < 0.01). The mean step-timing variabilities corresponding to before-sound, sound-on, and after-sound are 0.10, 0.05, and 0.08 s, respectively. Our results support the sound stimulation as an effective tool to improve gait performance, reflected by the suppression of *Lβ*/*Hβ*-HFO PAC, especially prominent for *Lβ*-HFO PAC, in the STN for patients with PD.

### Characteristics of *β*-Band–Related PAC During Stepping

**FIGURE 3:**
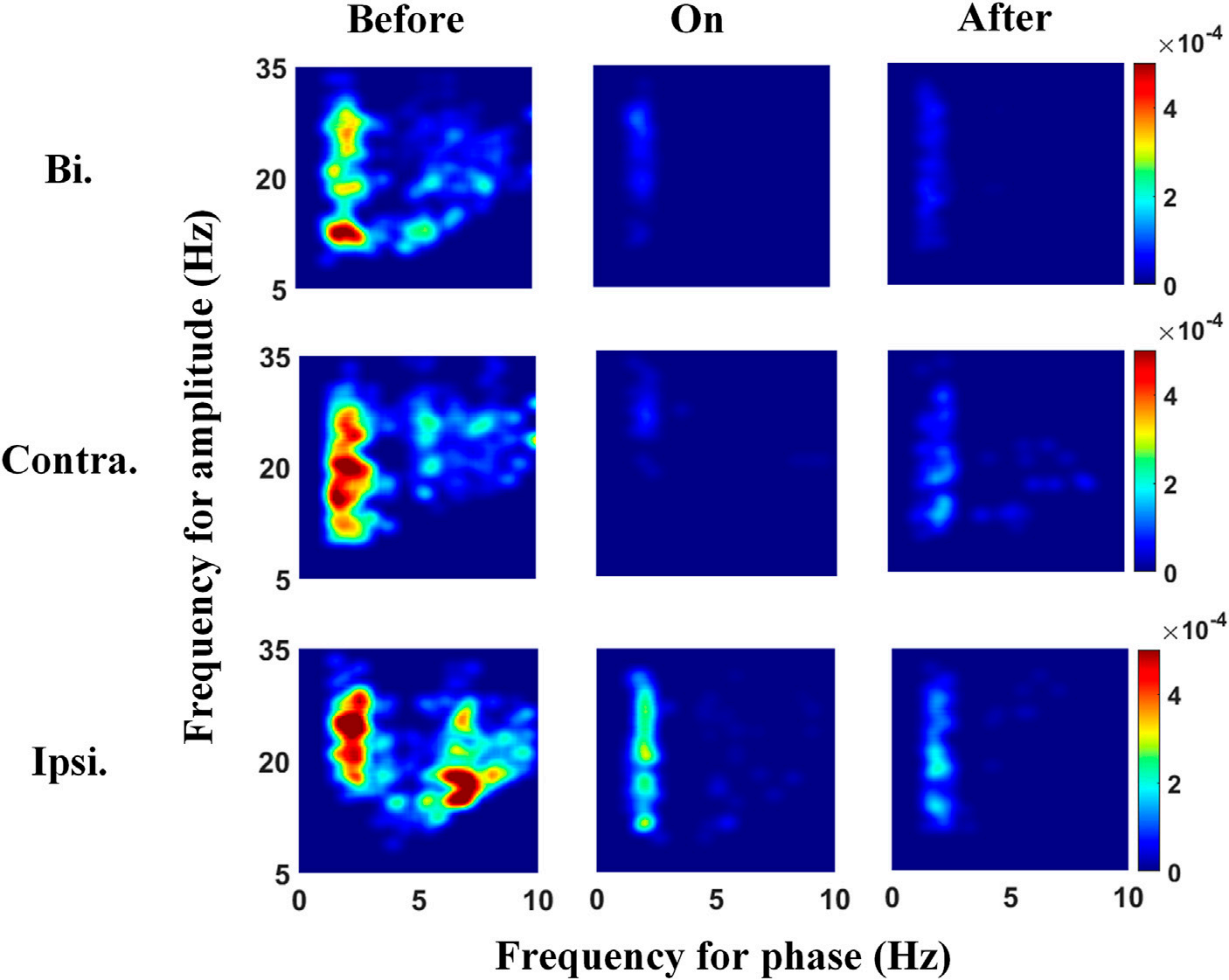
Phase-amplitude frequency planes under the three different sound conditions, from left to right corresponds to before, during, and after auditory cues. The top panels show the comodulograms reflecting the PAC across the whole 2-s gait cycle, and the middle panels link to the 1-s LFPs aligned to contralateral heel strike, whereas the bottom panels present the PAC around the 1-s ipsilateral heel strike. The strengths of *δ-Lβ/Hβ* PAC before providing auditory cues are higher than those after removing cues and are the smallest when applying cues. In addition, PAC around contralateral or ipsilateral heel strikes is higher than that of bilateral steps across three sound conditions. Bi, bilateral; Contra, contralateral; Ipsi, ipsilateral.

We explored the PAC between the *β* amplitude and its lower frequency phases in the STN to clarify whether the modulation of *β* power is susceptible to other frequency bands. The comodulograms in Figure 3 (left panels) show strong PAC between the *δ*-band and broadband-*β* (across *Lβ* and *Hβ*) activities before auditory cues. This *δ*-*β* PAC was suppressed when auditory cues were provided, followed by a slight rebound after cues, especially during contralateral steps. Meanwhile, the step-timing variability before, during, and after auditory cues corresponds to 0.26, 0.11, and 0.20 s, respectively. The abovementioned two findings infer that gait performance improved with sound stimulation, whilst this effect could preserve for a while after removing metronome sound, accompanied by decreased and resilient *δ*-*β* PAC. To find out whether the *δ-β* PAC is time-locked to the movement of contralateral steps or synchronous on both sides, we separated and re-linked all 1-s segmented LFPs around contralateral (middle panels in Figure 3) and ipsilateral (lowest panels in Figure 3) heel strikes, intending to distinguish the gait-related PAC. The contralateral PAC responds to sound stimulation the most, i.e., PAC was suppressed with cues and rebounded afterward, implying that *δ-β* PAC in the STN time-locks to the movement of steps. Of note, higher PAC was observed around contralateral and ipsilateral steps than that of the combination of bilateral steps.

### PAC in Other Frequencies During Stepping

**FIGURE 4:**
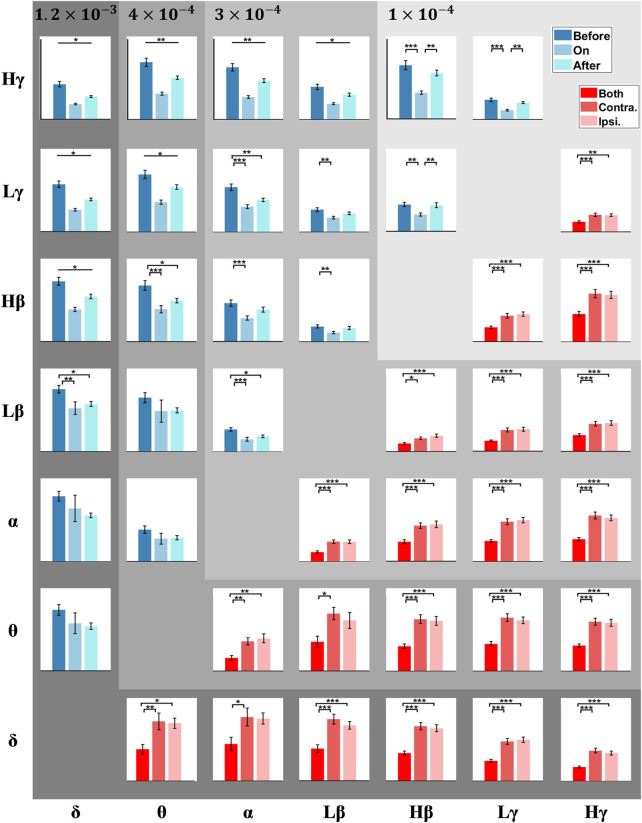
PAC matrix corresponds to different frequency pairs. Each box shows the PAC between a low-frequency phase and a high-frequency amplitude (e.g., the box at the topmost and leftmost position corresponds to the *δ-Hγ* PAC). The upper triangle of the matrix (blue) compares PAC before, during, and after auditory cues. The lower triangle of the matrix (red) compares PAC among bilateral, contralateral, and ipsilateral steps. The error bars represent the standard error of mean (SEM), and *, **, and *** correspond to *p* < 0.05, 0.01, and 0.001, respectively.

Except for the *δ*-*β* PAC, we further explored if PAC across other frequencies in the STN was involved, i.e., whether this PAC presents similar or opposite trends to the *δ*-*β* PAC under different sound conditions and step phases. To this end, GLMMs were executed, with the fixed factors including sound conditions (i.e., before-sound, sound-on, and after-sound) and step phases (i.e., bilateral, contralateral, and ipsilateral step), for the PAC between various STN neural decompositions in multiple frequencies. Figure 4 compares the PAC between all pair-wise STN-LFP components across varying states, of which the upper triangular matrix focuses on the effect of sound conditions, while the lower part emphasizes the timing of heel strikes. The statistical details are presented in Supplementary Table S3, wherein the significant *p*-values are emboldened. The sound stimulation seems to alter the variation of PAC in the STN for patients with PD during stepping, especially for the higher frequency amplitude-given (e.g., *β*/*γ*) PAC (i.e., the upper part of the upper triangular matrix in Figure 4), and such PACs were significantly suppressed with metronome sounds, and the effect could be faded partially after the withdrawal of auditory cues. As aforementioned, the mean step-timing variabilities corresponding to before-sound, sound-on, and after-sound are 0.10, 0.05, and 0.08 s, respectively.

On the other side, the exaggerated PAC of STN-LFPs around the 1-s unilateral steps, either for contralateral or ipsilateral steps, were significantly higher than that treating the combination of STN-LFPs around bilateral strikes as a whole (e.g., both the *δ-Lβ* PAC and *δ-Hβ* PAC present *p* < 0.0001). This fact prevails in all frequency pairs, especially for the *δ* and *θ* phase-given PAC, wherein the *Lβ*-band and *Hβ*-band amplitude-given PAC shows a slight trend of higher PAC during contralateral steps than that around ipsilateral steps.

### Phase Slips Between the Phase of High-Frequency Amplitude and Low-Frequency Phase in the STN During Stepping

**FIGURE 5:**
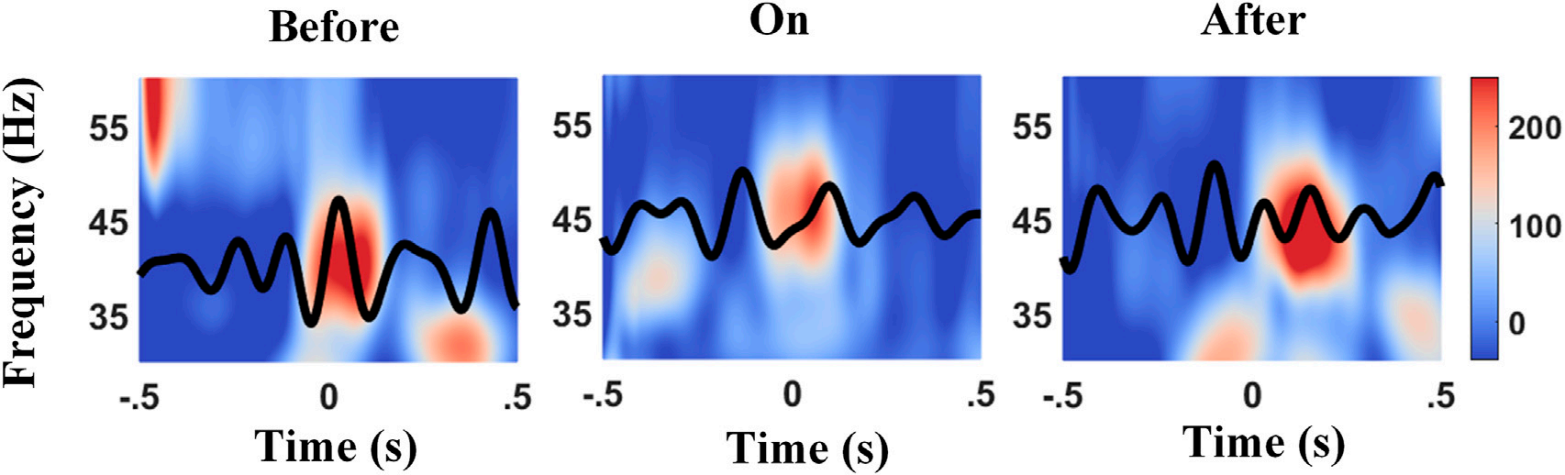
Example of phase slips between the *θ* phase and the peak of *Lγ* amplitude before, during, and after auditory cues. The time–frequency plot of the normalized power between 30–60 Hz (*Lγ*) was time-locked to the *θ* wave (black tracks), and 0 s aligns to the contralateral heel strikes. The peak of *Lγ* amplitude lies on the descending phase of the *θ* wave before auditory cues (left panel), in contrast, lies on the ascending phase of the *θ* wave with cues (middle panel), and lies the between after cues (right panel).

To explore whether the phase slips induce the diversity between bilateral PAC and contralateral/ipsilateral one, the phase shift between the phase of high-frequency amplitude and low-frequency phase was checked as well. The mean phase preference of individual 2-s epoch was calculated separately, based on the definition of PLV, followed by the averaged phase shift across all epochs of each channel as the output. Figure 5 presents an example showing the preference phases of the *θ* wave which aligns to the peak of the *Lγ* amplitude varied across different sound conditions. The local maximum of the *Lγ* amplitude lay on the descending phase of the *θ* wave before providing a metronome sound (left panel in Figure 5); in contrast, auditory cues altered the temporal structure with the *Lγ* amplitude peaked at the ascending phase of the *θ* wave (middle panel in Figure 5), whereas the phase slip between the *θ* phase and the phase of *Lγ* amplitude fell in between after cues (right panel in Figure 5), implying that auditory stimulation might force a displacement of the preferred *θ* phase for *Lγ* activity (López-Azcárate et al., 2013).

### The Impact of Finer Gait Phases on PLVs for STN-LFP

**FIGURE 6:**
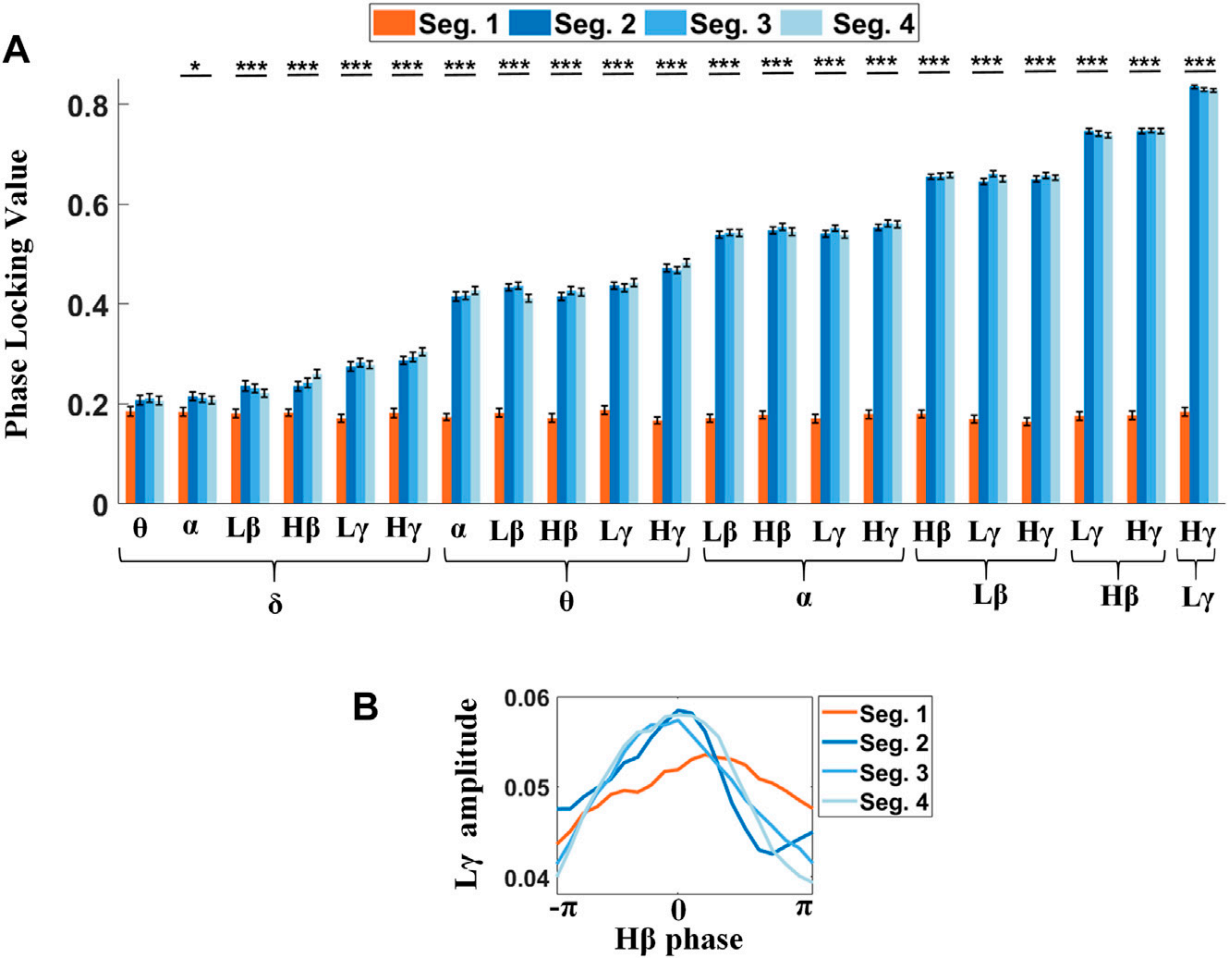
PLVs between the low-frequency phase and the phase of high-frequency amplitude are sensitive to step phases. **(A)** Significant differences reveal in almost all PLVs across step phases, wherein only the *δ-θ* PLVs show no significant difference. **(B)** Phase shifts between the *Hβ* phase and *Lγ* amplitude for four different segments (Seg. 1: contralateral heel strike, Seg. 2: the contralateral foot stand, Seg. 3: ipsilateral heel strike, and Seg. 4: ipsilateral foot stand) are shown in the phase-amplitude plot. The orange line shows the phase shift around the contralateral step (Seg. 1), wherein the *Lγ* activity reaches its peak while the *Hβ* phase is descending. However, in the Seg. 2-4, the *Lγ* amplitude modulations keep pace with the *Hβ* phase (blue lines).

Next, we further explored the dynamics of PLVs in the STN during a gait cycle with finer segmentation. Each 2-s complete gait cycle was parsed into four 0.5-s phases (Figure 6), including the contralateral heel strike (Seg. 1), the contralateral foot stand (Seg. 2), the ipsilateral heel strike (Seg. 3), and the ipsilateral foot stand (Seg. 4), respectively, in chronological order. The degree of phase synchronization between the low-frequency wave and high-frequency amplitude was estimated by the magnitude of PLV, wherein the angular unit is the radian. As shown in Figure 6A and Supplementary Table S4, PLVs around contralateral heel strikes (Seg. 1) were significantly lower than the rest (Seg. 2 to Seg. 4) in almost all pairs of frequency bands except for *δ-θ* PLV (F = 1.84, *p* = 0.1385), whilst among the remaining pairs of frequency bands, almost all PLVs showed the *p*-value < 0.0001 except for *δ-α* PLV (F = 2.71, *p* = 0.0443), indicating that contralateral heel strikes suppressed the PAC, thus resulting in a misalignment between the low-frequency phase and the phase of high-frequency amplitude in the STN, especially prominent for the higher frequency phase-given components (Figure 6A and Supplementary Table S4). Meanwhile, there’s a clear trend of rising PLVs in Seg. 2 to 4, from ∼0.2 to ∼0.8, which correlated with the frequencies of the involved oscillators for PLVs, wherein the phase-given components (e.g., *δ*, *θ*, *α*, *Lβ*, *Hβ*, and *Lγ*) contributed more to this difference than the amplitude-given ones (e.g., *θ*, *α*, *Lβ*, *Hβ*, *Lγ*, and *Hγ*).

The significance tests of the phase shifts between different pairs of frequency components on the step phases showed that PLVs around contralateral heel strikes differed from the others. Figure 6B is an example showing that the *Lγ* amplitude tended to be misaligned to the *Hβ* phase with a lower peak value around contralateral heel strikes (orange line), as compared to the other three step phases (blue lines), wherein a smoothed envelope of the *Lγ* amplitude histogram over the *Hβ* phase was applied.

At last, we examined whether the influence of sound stimulation on the changes in PLVs in the STN varied by the step phases (Supplementary Figure S1). Most PLVs present significant differences to sound conditions around contralateral heel strikes (Seg. 1), wherein such PLVs were suppressed with cues and then rebounded if they were withdrawn (Supplementary Figure S1A). The trend was the same as that of bilateral PAC. Interestingly, only the *δ*-band-related PLVs tended to reveal significant differences across various sound conditions for the rest of the three step phases (Supplementary Figure S1B–D). Of note, the correlations between STN-PLVs and the frequencies of involved LFP components resembled those of Figure 6A.

### High-*β* Power Modulation in the STN Is Sensitive to Sound Stimulation During Stepping

**FIGURE 7:**
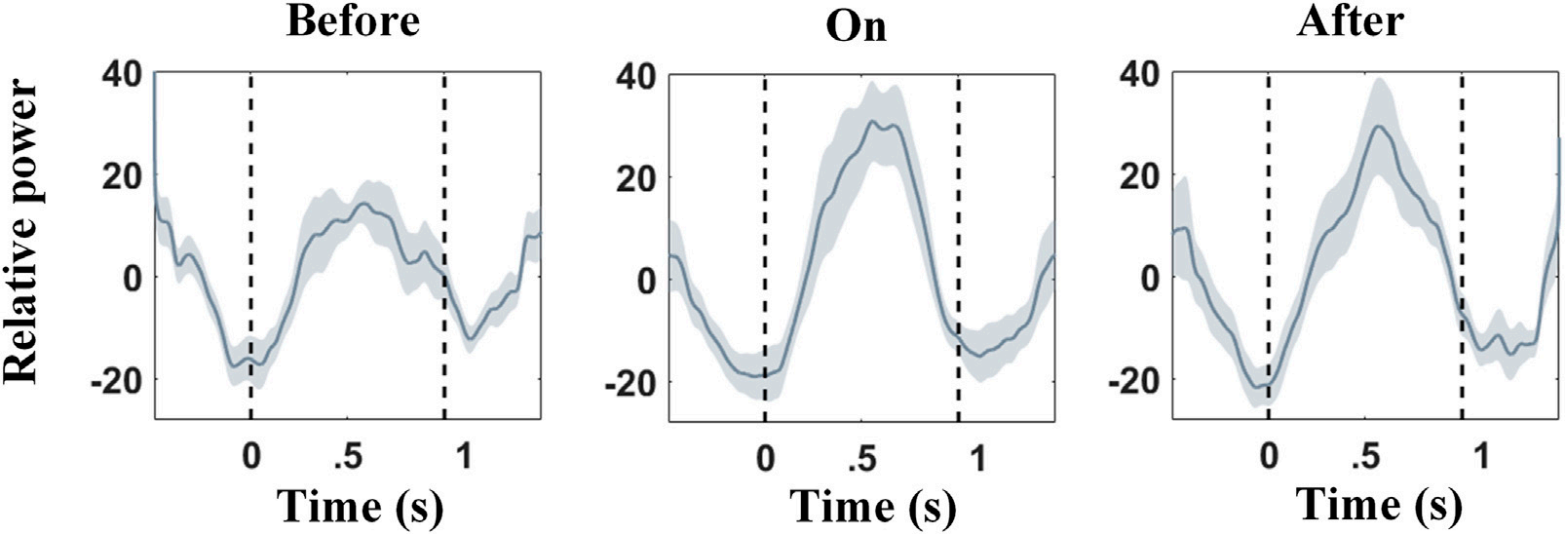
Relative *Hβ* power modulations across subjects under three different sound conditions (before, during, and after auditory cues). 0 s aligns to the contralateral heel strikes, while 1 s aligns to the ipsilateral heel strikes. Shaded areas denote the standard error (SE). *Hβ* relative power is reduced around contralateral heel strike, followed by a rebound afterward. This effect can be strengthened with auditory cues.

*β* activity has long been thought to play an important role for patients with PD. Our results support that *Hβ* power modulates greater than that of the *Lβ*. The normalized *Hβ* power modulation was calculated under different sound conditions per 2-s epoch, spanning from 0.5 s before a contralateral heel strike to 0.5 s after the next ipsilateral heel strike, and then averaged across all epochs as the output. In Figure 7, the first trough of *Hβ* power modulation aligned to the timing of contralateral heel strikes and then rose to a peak in the mid of two strikes (i.e., contralateral foot stand); next, the second trough appeared shortly after the ipsilateral heel strike (not perfectly aligned). To confirm that the *Hβ* power modulation was stronger with sound stimulations, pairwise power modulations were subjected to the Wilcoxon signed-rank test. Power modulations were computed as the maximum power minus the minimum power of *Hβ* band modulation within 0–1 s in the stepping cycle (between contralateral heel strike and ipsilateral heel strike in Figure 7). Statistical results showed that *Hβ* power modulation was enlarged with the support of auditory cues (*n* = 9 and *p* = 0.006), and such an effect can be preserved for a while (*n* = 9 and *p* = 0.26).

### Phase Synchrony Between STN-LFP Frequency Modulation and the Gait Phase

**FIGURE 8:**
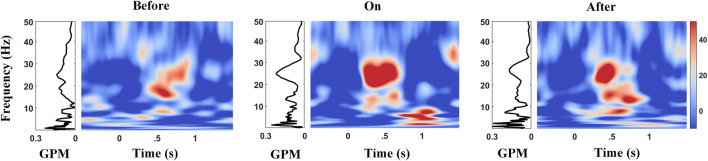
Amplitude modulations in multiple frequencies of STN-LFPs during gait with the corresponding GPM values before, during, and after auditory cues. The time–frequency (TF) plot shows amplitude modulations in the STN vary across a gait cycle, 0 s aligns to the contralateral heel strikes. Diagrams on the left of TF plots show the corresponding GPM spectrums up to 50 Hz, wherein *Hβ* and low frequency (<4 Hz) reveal high GPM values.

**FIGURE 9:**
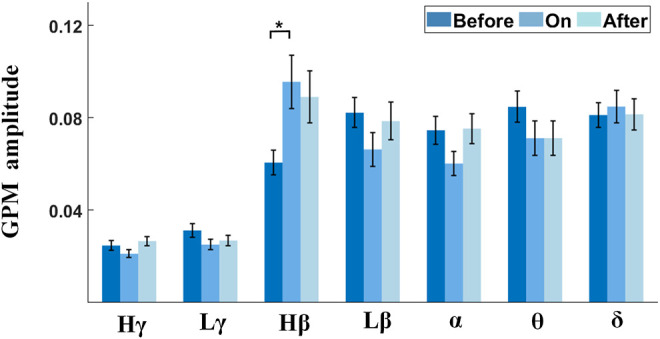
Comparisons of GPM amplitudes in multiple frequency bands across different sound conditions. Gait-phase–related modulations across various sound conditions are grouped by different frequency bands of STN-LFP. Only the *Hβ* frequency band shows a significant difference between before-sound and sound-on conditions.

Studies reported that the gait phase was shown to come with apparent *α*/*β* modulations in PPN for PD (He et al., 2021). To quantify the strength of amplitude modulation in multiple frequencies (STN-LFPs) time-locked to gait, we calculated the GPMs between STN-LFP frequency modulation and the gait phase. The right panel of each subplot in Figure 8 (color plot) shows the averaged scalogram of STN-LFPs across 2-s gait cycles, wherein 0 corresponds to the time points of contralateral heel strikes, and the left panel of each subplot presents the GPM spectrums up to 50 Hz. Interestingly, pronounced *Hβ* modulation relative to a gait cycle appeared with the metronome sound (Figure 8, middle subplot), as compared to that before providing cues (Figure 8, left subplot), rising to a local maximum around 0.5 s after contralateral heel strikes, along with the corresponding GPM that reached to a local maximum value around ∼25 Hz (F = 4.56, *p* = 0.0117; see Figure 9 and Supplementary Table S5). Similarly, this elevated *Hβ*-GPM (or the pronounced *Hβ* synchronization) was partially reduced after the metronome sound was removed (Figure 8, right panel). In addition, the *δ* wave (<4 Hz) also presented high GPM values (left panels of subplots in Figure 8) due to a relatively stronger phase coherence between a low-frequency modulation and a standard sine wave, even though the frequency power *per se* was lower than the *Hβ* activity (right panels of subplots in Figure 8). Of note, the *δ*-GPM prevailed across all sound conditions (F = 0.10 and *p* = 0.9061; see Figure 9 and Supplementary Table S5).

## Discussion

### Pathological Role of PAC During Stepping for PD

de Hemptinne et al. (2015) reported that the *β* phase modulates broadband-*γ* amplitude (50–200 Hz) in M1 for PD patients at rest, whereas this excessive *β-γ* PAC can be suppressed by DBS or dopaminergic medications (Swann et al., 2015). Later, van Wijk et al. associated the *β* phase to an even higher frequency oscillation (i.e., HFO: 150–400 Hz) at rest and correlated *β*-HFO PAC in the STN with UPDRS III motor subscores (bradykinesia/rigidity), prevailing across *Lβ* (*r* = 0.33, *p* = 0.001) and *Hβ* activities (*r* = 0.21, *p* = 0.039). These studies infer that the *β* phase could modulate broadband-*γ*/HFO amplitude for PD patients at rest (de Hemptinne et al., 2013; Yang et al., 2014; de Hemptinne et al., 2015; Swann et al., 2015; van Wijk et al., 2016), either in STN or M1. In this work, abnormal *β*-*γ*/HFO PAC in the STN for PD patients during gait was further revealed and verified. This excessive *β-γ*/HFO PAC in the STN was linked with motor impairment in gait for PD patients, wherein such abnormal STN *β-γ*/HFO PAC was suppressed with a rhythmic auditory cue, along with an improvement in gait performance, followed by a slight rebound of STN *β-γ*/HFO PAC shortly after removing the metronome sound (*Lβ-Lγ* PAC: *p* = 0.0031; *Lβ-Hγ* PAC: *p* < 0.0001; *Hβ-Lγ* PAC: *p* = 0.0012; *Hβ-Hγ* PAC: *p* < 0.0001; *Lβ*-HFO PAC: *p* < 0.0001; *Hβ*-HFO PAC: *p* = 0.0020). One intriguing explanation for the prominent linkage between the suppression of *β-γ* PAC in the STN and motor improvement in gait might be a reductive constraint on prokinetic *γ* amplitude modulation from the *β*-band phase.

In our results, PAC between *γ* amplitude (*Lγ*- and *Hγ*-band) and its lower-frequency phases (i.e., *δ*, *θ*, *α*) also presents significant differences across sound conditions. The PAC mentioned earlier is negatively correlated with gait performance. Our results imply that motor impairment in gait involves multiple phase-given oscillators entangled with *γ*-band activities. Once the constraint to the *γ*-band amplitude caused by broadband low-frequency phases lessens, the *γ*-band modulation could be greatly liberated. Thus, the movement-related *γ* modulation was restored.

Meanwhile, PAC between *β*-band amplitude modulation and its lower-frequency phases (i.e., *δ*, *θ*, *α*) presented trends similar to that of the *γ*-band (*δ-Lβ* PAC: *p* = 0.0034; *θ-Lβ* PAC: *p* = 0.2627; *α-Lβ* PAC: *p* < 0.0001; *δ-Hβ* PAC: *p* < 0.0001; *θ-Hβ* PAC: *p* < 0.0001; and *α-Hβ* PAC: *p* = 0.0008), indicating that abnormal PAC in the STN for PD patients during gait might not necessarily directly link with *γ*-band oscillations. One possible explanation might be the constraints from low-frequency phases on the *β*-band activity are reduced, thus relieving the *β*-band amplitude modulation. Of note, the *β*-band amplitude modulation, which is different from the concept of *β*-band power, positively correlates with motor improvement in gait (Fischer et al., 2018). Our findings further support that *β*-band–related PAC could be a set of effective biomarkers for PD patients while stepping and intervening by auditory cues.

### Critical Phase During Stepping Reflective to Dynamics in PAC

Our results showed that the PAC around contralateral and/or ipsilateral heel strikes was higher than that of the combination of both in general (see the lower triangular matrix in Figure 4). Despite no significant phase slips in PAC around the individual side, a relatively uniform phase-amplitude distribution resulting from the diverse phase preferences between contralateral and ipsilateral PAC might be the main cause. Several previous reports support the PAC in BG matter to the control of unilateral movement. In globus pallidus interna (GPi), only the contralateral GPi presents attenuated *Lβ-Lγ* and *β*-HFO PAC when executing unilateral movement (AuYong et al., 2018). In the STN, contralateral *θ-γ* PAC emerges right after a voluntary muscle contraction for PD patients (Kato et al., 2016), whilst *β*-HFO PAC is significantly higher in the clinically more affected side at rest (Shreve et al., 2017).

More precisely, the coupling strength around contralateral heel strike (Seg. 1) with approximately the same levels across all frequency pairs was significantly lower than that of the other three step phases (Figure 6A and Supplementary Table S4), of which the differences were especially prominent for PAC-involved higher-frequency phase-given oscillations. Interestingly, only the PLVs around the contralateral heel strike (Seg. 1) were sensitive to metronome sound (Supplementary Figure S1A), implying contralateral heel strike as a critical movement facilitates sound stimulation. Our result in the suppression of PAC in the STN along with contralateral limb movement resembles past finding which revealed unilateral upper limb movement attenuates *Lβ-Lγ* and *β*-HFO PAC in contralateral GPi for PD patients (AuYong et al., 2018). A similar change in PAC seems to prevail across the BG-M1 circuit, e.g., PAC in M1 and inter-region PAC between the thalamus (phase-given) and M1 (amplitude-given) both revealed more clear suppressed coupling strength on the contralateral than the other side (Opri et al., 2019). All support contralateral footsteps, especially the contralateral heel strike, are associated with changes in PACs. One intriguing inference might be that contralateral heel strike breaks the blockage in the neural circuit for effective external auditory stimulations, enabling rhythmic metronome sounds to dissociate the potential pathological PAC in the STN. Given that contralateral step facilitated auditory stimulation, it seems that intermittent stimulation strategies (i.e., auditory cues time-locked to the contralateral step) may be superior to constant stimulation for improving gait in PD.

### Phase Slips Between the Low-Frequency Wave and High-Frequency Amplitude Shift by Step Phases

The phase-locking values (PLVs) between the low-frequency phase and the phase of high-frequency amplitude monotonically rise when the frequency of the phase-given component increases during steady-step phases (Seg. 2, Seg. 3, Seg. 4 in Figure 6A). The consistency in phase differences determines the PLVs. One possible inference might be the high-frequency components including *γ*-band and *β*-band oscillations, which are of critical roles for movement and parkinsonism, respectively. Thus, high PLVs between *β* activity and *γ* amplitude constrain modulation of *γ* amplitude, as previously stated.

In addition, despite the quite different PLVs among different frequency pairs in the steady-step phases, contralateral heel strike (Seg. 1) dissociated the time lock between the low-frequency phase and high-frequency amplitude, resulting in the approximate same levels in PLVs, as shown in Figure 6A. Therefore, we hypothesized an increasing misalignment in phases between the low-frequency activity and high-frequency amplitude around the timing of contralateral heel strikes (Seg. 1) relative to the other three steady-step phases, and Figure 6B shows *Lγ*-band amplitude preceded the *Hβ*-band phase, while the phase slips during the other three steady-step phases close to zero with more centralized phase-amplitude distribution patterns, as expected.

### Role of STN *β*-band Amplitude Modulation During Stepping for PD

*β*-band power of LFP in the STN correlates with akinetic/rigid motor impairment for patients with PD, whereas interventions including DBS and levodopa medication attenuate the excessive *β*-band synchronization along with improvement in motor performances (Uhlhaas and Singer, 2006; Hammond et al., 2007; Kühn et al., 2008). Several studies reported dopaminergic medication ameliorates the symptoms (especially bradykinesia, rigidity, and/or tremor) of PD, reflected by increasing *β-*band event-related desynchronization (ERD) (Doyle et al., 2005; Hanrahan et al., 2016). Similarly, auditory cue improves performance in finger tapping (i.e., less time difference between real tapping and cue) (Bichsel et al., 2018) along with increased *β*-band ERD. Nevertheless, whether *β-*band amplitude modulation correlates with such *β*-band ERD remained unclear.

As te Woerd et al. (2014) reported predictive cue enlarges *β*-band modulation, implying assistive external cueing that may bring facilitatory effects in *β*-band modulation depth. The *Hβ*-band modulation increases in the STN for PD patients while bicycling compared to simple walking, of which the former eliminates the occurrence of freezing gait (Storzer et al., 2017). Also, temporal *β* reactivity negatively correlates with UPDRS III motor subscores (Little et al., 2012). In light of the aforementioned reports, we hypothesized that rhythmic sound stimulation increases *β-*band modulation, especially *Hβ*-band amplitude modulation in the STN for PD patients, and facilitates normal movement patterns in stepping.

To clarify this point, in our result, GPM was shown to significantly differ across sound conditions for *Hβ*-band oscillations (Figure 9 and Supplementary Table S5). Briefly, metronome sound induces an enhanced *Hβ*-band GPM along with improvement in stepping, reflected by decreased step-timing variability (Figure 8). This finding implies not only the exaggerated *β* synchronization in general but also the insufficient amplitude modulation of the *β*-band activity are signs of motor impairment for PD (Brittain and Brown, 2014).

### GPM in a Low-Frequency Band

When calculating the amplitude modulation strength in the STN over the gait phase with the GPM method, not only the *Hβ*-band but also low-frequency components (<4 Hz) presented high GPM amplitude levels (local maxima). Since the magnitude of GPM resembles the correlation between the oscillatory envelope at a certain frequency band and a sinusoid reconstructed by whole gait phases, reflecting modulation of the oscillatory amplitude is relative to a gait cycle, and the GPM magnitude equals 1 (maxima), while brain signal modulates sinusoidally time-locked to step cycles. Based on the definition of GPM, the low-frequency amplitude modulation distorts less by the same disturbance, and thus the waveform, in general, resembles more the reconstructed sinusoid with a step frequency, thus revealing a higher GPM magnitude over the low-frequency range (<4 Hz), as shown in Figure 8. In all, MI measures whether the accumulated amplitude is confined within a certain range of phases, while GPM emphasizes more if amplitude modulation resembles or is regulated by gait/step phases.

### Limitations to This Study

The LFPs in this study were recorded from the bilateral DBS electrodes implanted in the STN. Therefore, contributions to the PAC in the STN from the basal ganglia motor circuits, e.g., GPi, etc., are beyond our scope. Our results reveal the critical role of the multiple nesting interactions and *Hβ* modulation in the STN-LFP under gait impairment, as well as their changes with cues. However, whether these findings are useful in neurofeedback training or predicting gait require further validation. Also, whether these findings prevail in the combined use of cues, such as interactive video games is unknown (Alhasan et al., 2022).

Another limitation to this study is that the LFPs were recorded from seated patients while stepping, instead of walking upright. The fact whether our findings also occur in the case of free walking is beyond our scope. However, past studies showed similar modulations and time–frequency features between stepping when sitting and free walking (Fischer et al., 2018; He et al., 2021), which supports the generalizability of our results to real gait.

The number of patients is limited due to the invasive nature of STN-DBS, wherein the LFPs collected with information while stepping are rare in particular. Thus, a further limitation to this study is that our present findings may require more recordings (i.e., a larger sample size) as validation.

## Conclusion

In PD, auditory cues improve stepping performance, which went along with the suppression of exaggerated *β*/*γ*-band-related PAC and an enhanced gait-phase–related *Hβ* modulation in the STN. In addition, such PAC values (or PLVs) were suppressed to a certain level which was sensitive to sound stimulation when the contralateral foot fell. Our results indicate that MPAC is a useful tool in estimating neural interactions, and both *β*/*γ*-band-related PAC and/or *Hβ* modulation are linked to gait phases (or STN-*Hβ* GPM) associated with sound stimulation.

## Data Availability

The original contributions presented in the study are included in the article/Supplementary Material, further inquiries can be directed to the corresponding author.
