# Supplementary material for: Frequency Nesting Interactions in the Subthalamic Nucleus Correlate With the Step Phases for Parkinson’s Disease
Source: Front Physiol. 2022 Apr 29;13:890753. doi: 10.3389/fphys.2022.890753 (PMC9100409; doi:10.3389/fphys.2022.890753)
Supplement: Supplementary file 1 [file DataSheet1.PDF]

Supplementary Table 1. Demographic and clinical information of all subjects.

Sub. ID	Age	Sex	Handed-ness	Main Symptom	Disease Duration (yrs)	UPDRS-III OFF/ON levodopa	DBS Lead	Sound Condition
1	62	M	R	FOG	12	27/4	Medtronic 3389	Off
2	60	M	R	FOG	8	52/30	Medtronic 3389	Off
3	59	M	R	tremor, bradykinesia, dyskinesia	7	53/18	Boston Scientific DB-2202	Off/On
4	64	F	R	rigidity, tremor, FOG	16	66/36	Boston Scientific DB-2202	Off/On
5	59	M	R	fluctuations, tremor	14	36/8	Medtronic 3389	Off/On
6	56	M	L	fluctuations, dyskinesia	7	42/26	Medtronic 3389	Off/On
7	62	M	R	tremor, rigidity, dyskinesia, mild FOG	12	59/15	Medtronic 3389	Off/On
8	71	M	R	tremor, FOG	15	36/18	Boston Scientific DB-2201	Off/On
9	61	M	R	rigidity	9	33/11	Medtronic 3389	Off/On
10	57	M	R	tremor, FOG	12	49/18	Boston Scientific DB-2201	Off/On
11	59	M	R	tremor, mild FOG	10	28/8	Boston Scientific DB-2201	Off/On
12	59	M	R	fluctuations, mild FOG	22	23/6	Medtronic 3389	Off
13	65	M	R	tremor	8	16/8	Medtronic 3389	Off

F, female; M, male; L, left; R, right; FOG, freezing of gait; Off, sound off; On, sound on.

Supplementary Table 2. Significant test for the β-HFO PACs with sound conditions (three levels, Before, On and After) as the fixed factor.

Index	df	SS	MS	F-Value	P-Value
$L\beta$-HFO	2	6.61e-8	3.30e-8	12.42	**<.0001**
$H\beta$-HFO	2	2.12e-8	1.06e-8	6.42	**0.0020**

Significant *p*-values are emboldened.

Supplementary Table 3. Statistical results for PACs in twenty-one different frequency pairs. Fixed factors include sound (three levels, Before, On and After) and lateral (three levels, Bi., Contra. and Ipsi.) conditions.

PAC	Factor	df	SS	MS	F-Value	P-Value
δ - θ	Sound	2	7e-6	0.0000035	1.9136	0.1485
	Lateral	2	2.03e-5	0.0000101	5.5410	**0.0041**
	Sound*Lateral	4	2.19e-6	5.4728e-7	0.2990	0.8786
δ - α	Sound	2	8.18e-6	4.0905e-6	1.7070	0.1824
	Lateral	2	1.80e-5	9.0043e-6	3.7576	**0.0239**
	Sound*Lateral	4	2.44e-6	6.1055e-7	0.2548	0.9067
δ - $L\beta$	Sound	2	9.24e-6	4.60e-6	5.75	**0.0034**
	Lateral	2	2.11e-5	1.05e-5	13.13	**<.0001**
	Sound*Lateral	4	1.42e-6	3.56e-7	0.44	0.7777
δ - $H\beta$	Sound	2	1.73e-5	8.63e-6	19.83	**<.0001**
	Lateral	2	1.88e-5	9.38e-6	21.55	**<.0001**
	Sound*Lateral	4	2.91e-6	7.28e-7	1.67	0.1549
δ - $L\gamma$	Sound	2	1.50e-5	7.49e-6	31.44	**<.0001**
	Lateral	2	1.15e-5	5.77e-6	24.21	**<.0001**
	Sound*Lateral	4	1.28e-6	3.20e-7	1.34	0.2531
δ - $H\gamma$	Sound	2	9.05e-6	4.53e-6	30.35	**<.0001**
	Lateral	2	6.30e-6	3.15e-6	21.12	**<.0001**
	Sound*Lateral	4	1.16e-6	2.89e-7	1.94	0.1027
θ - α	Sound	2	2.46e-7	1.23e-7	1.76	0.1737
	Lateral	2	1.02e-6	5.10e-7	7.28	**0.0008**
	Sound*Lateral	4	2.18e-7	5.45e-8	0.78	0.5403
θ - $L\beta$	Sound	2	5.63e-7	2.81e-7	1.34	0.2627
	Lateral	2	1.73e-6	8.64e-7	4.11	**0.0169**
	Sound*Lateral	4	7.90e-7	1.98e-7	0.94	0.4399
θ - $H\beta$	Sound	2	1.44e-6	7.21e-7	9.98	**<.0001**
	Lateral	2	2.10e-6	1.05e-6	14.54	**<.0001**
	Sound*Lateral	4	3.19e-7	7.98e-8	1.10	0.3536

θ - Lγ	Sound	2	1.87e-6	9.33e-7	18.36	**<.0001**
	Lateral	2	1.96e-6	9.81e-7	19.31	**<.0001**
	Sound*Lateral	4	1.74e-7	4.34e-8	0.86	0.4908
θ - Hγ	Sound	2	2.40e-6	1.20e-6	29.71	**<.0001**
	Lateral	2	1.67e-6	8.37e-7	20.71	**<.0001**
	Sound*Lateral	4	1.77e-7	4.43e-8	1.10	0.3579
α- Lβ	Sound	2	1.43e-7	7.16e-8	9.38	**<.0001**
	Lateral	2	1.85e-7	9.27e-8	12.16	**<.0001**
	Sound*Lateral	4	3.13e-8	7.84e-9	1.03	0.3923
α- Hβ	Sound	2	3.08e-7	1.54e-7	7.26	**0.0008**
	Lateral	2	5.02e-7	2.51e-7	11.85	**<.0001**
	Sound*Lateral	4	6.38e-8	1.59e-8	0.75	0.5568
α- Lγ	Sound	2	5.57e-7	2.79e-7	15.82	**<.0001**
	Lateral	2	6.99e-7	3.40e-7	19.85	**<.0001**
	Sound*Lateral	4	5.99e-8	1.50e-8	0.85	0.4940
α- Hγ	Sound	2	1.19e-6	5.94e-7	30.19	**<.0001**
	Lateral	2	8.68e-7	4.34e-7	22.08	**<.0001**
	Sound*Lateral	4	1.29e-7	3.21e-8	1.63	0.1642
Lβ- Hβ	Sound	2	5.07e-8	2.54e-8	4.94	**0.0075**
	Lateral	2	7.63e-8	3.82e-8	7.42	**0.0007**
	Sound*Lateral	4	1.93e-8	4.84e-9	0.94	0.4399
Lβ- Lγ	Sound	2	8.41e-8	4.21e-8	5.84	**0.0031**
	Lateral	2	2.16e-7	1.08e-7	15.00	**<.0001**
	Sound*Lateral	4	3.54e-8	8.84e-9	1.23	0.2984
Lβ- Hγ	Sound	2	3.84e-7	1.92e-7	16.33	**<.0001**
	Lateral	2	2.43e-7	1.22e-7	10.34	**<.0001**
	Sound*Lateral	4	3.01e-8	7.53e-9	0.64	0.6341
Hβ- Lγ	Sound	2	1.76e-8	8.78e-9	6.81	**0.0012**
	Lateral	2	3.17e-8	1.59e-8	12.30	**<.0001**
	Sound*Lateral	4	1.34e-8	3.35e-9	2.60	**0.0354**

$H\beta$- $H\gamma$	Sound	2	1.16e-7	5.82e-8	14.98	**<.0001**
	Lateral	2	7.35e-8	3.67e-8	9.46	**<.0001**
	Sound*Lateral	4	1.67e-8	4.18e-9	1.08	0.3674
$L\gamma$ - $H\gamma$	Sound	2	1.63e-8	8.14e-9	16.32	**<.0001**
	Lateral	2	9.24e-9	4.62e-9	9.27	**0.0001**
	Sound*Lateral	4	7.75e-10	1.94e-10	0.39	0.8169

Significant *p*-values are emboldened.

Supplementary Table 4. Significant tests for PLVs between different pairs of frequency bands with the step-phases as the fixed factor (four levels, Seg. 1, Seg. 2, Seg. 3 and Seg. 4).

Pair	df	SS	MS	F-Value	P-Value
δ - θ	3	0.08	0.03	1.84	0.1385
δ - α	3	0.11	0.04	2.71	**0.0443**
δ - $L\beta$	3	0.36	0.12	8.33	**<.0001**
δ - $H\beta$	3	0.62	0.21	15.35	**<.0001**
δ - $L\gamma$	3	1.64	0.55	39.40	**<.0001**
δ - $H\gamma$	3	1.82	0.61	41.86	**<.0001**
θ - α	3	8.55	2.85	244.19	**<.0001**
θ - $L\beta$	3	8.53	2.84	250.96	**<.0001**
θ - $H\beta$	3	8.83	2.94	239.22	**<.0001**
θ - $L\gamma$	3	8.75	2.92	258.45	**<.0001**
θ - $H\gamma$	3	13.26	4.42	459.42	**<.0001**
α - $L\beta$	3	19.18	6.39	699.10	**<.0001**
α - $H\beta$	3	19.30	6.43	684.03	**<.0001**
α - $L\gamma$	3	19.54	6.51	651.33	**<.0001**
α - $H\gamma$	3	20.16	6.72	685.95	**<.0001**
$L\beta$ - $H\beta$	3	31.88	10.63	1390.18	**<.0001**
$L\beta$ - $L\gamma$	3	32.69	10.90	1287.51	**<.0001**
$L\beta$ - $H\gamma$	3	33.63	11.21	1639.12	**<.0001**
$H\beta$ - $L\gamma$	3	44.92	14.97	2104.43	**<.0001**
$H\beta$ - $H\gamma$	3	45.53	15.18	2217.08	**<.0001**
$L\gamma$- $H\gamma$	3	58.63	19.54	4243.11	**<.0001**

Significant *p*-values are emboldened.

Supplementary Table 5. Significant tests for GPM amplitude of STN-LFP in different frequency bands with the sound conditions (three levels, Before, On and After) as the fixed factor.

Index	df	SS	MS	F-Value	P-Value
δ	2	4.88e-4	2.44e-4	0.10	0.9061
θ	2	8.32e-3	4.16e-3	1.29	0.2786
α	2	8.43e-3	4.21e-3	1.85	0.1609
$L\beta$	2	8.43e-3	4.21e-3	1.29	0.2773
$H\beta$	2	4.66e-2	2.33e-2	4.56	**0.0117**
$L\gamma$	2	1.33e-3	6.64e-4	1.51	0.2245
$H\gamma$	2	8.56e-4	4.28e-4	1.68	0.1888

Significant *p*-values are emboldened.

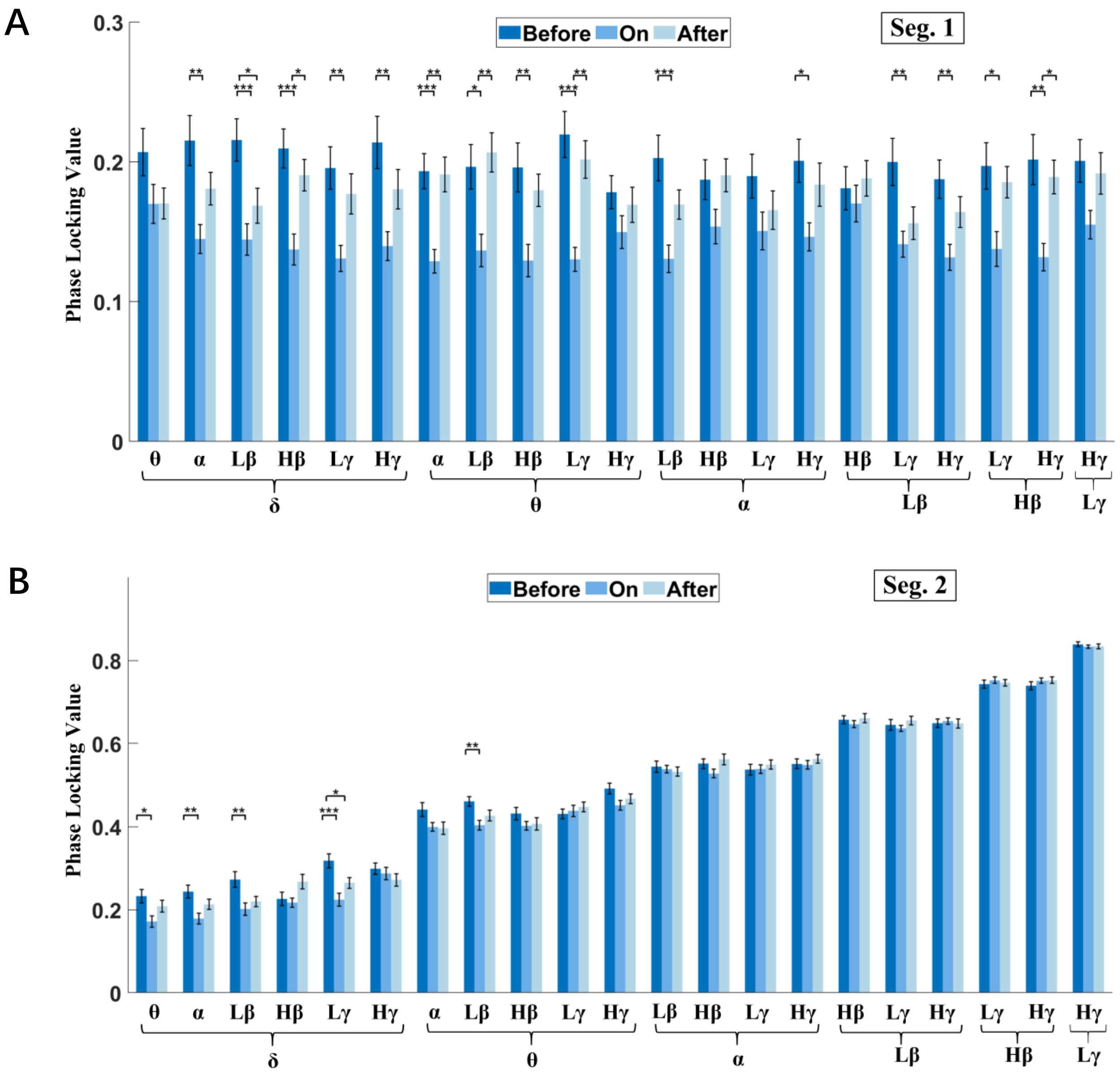
A
Before On After
Seg. 1
Phase Locking Value
0.3
0.2
0.1
0
θ α Lβ Hβ Lγ Hγ α Lβ Hβ Lγ Hγ Lβ Hβ Lγ Hγ Hβ Lγ Hγ Lγ Hγ Hγ
δ θ α Lβ Hβ Lγ
B
Before On After
Seg. 2
Phase Locking Value
0.8
0.6
0.4
0.2
0
θ α Lβ Hβ Lγ Hγ α Lβ Hβ Lγ Hγ Lβ Hβ Lγ Hγ Hβ Lγ Hγ Lγ Hγ Hγ
δ θ α Lβ Hβ Lγ

C

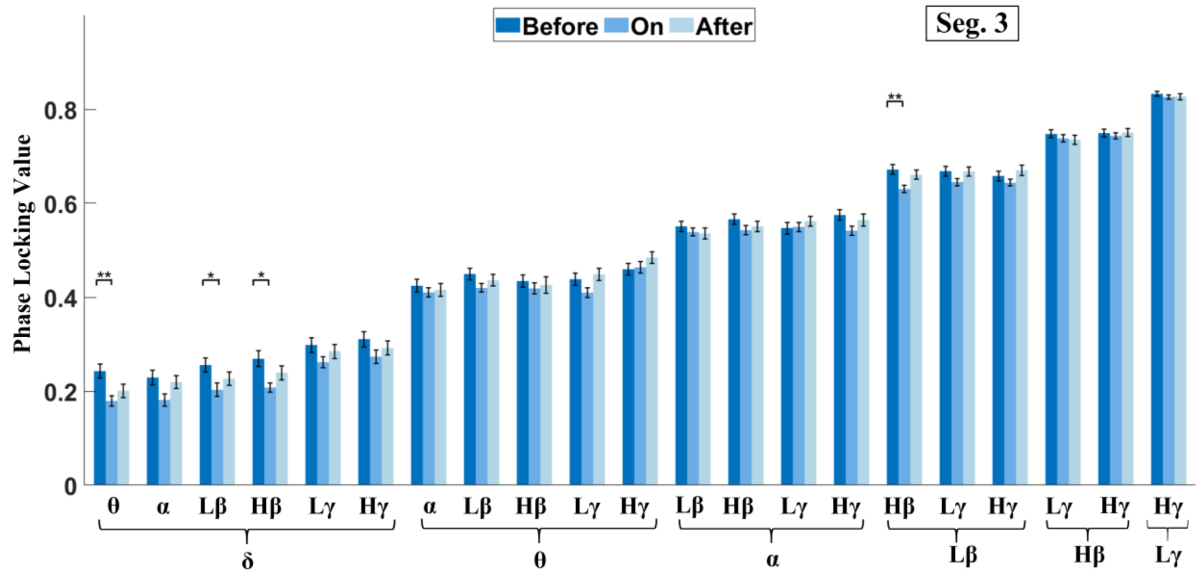

D

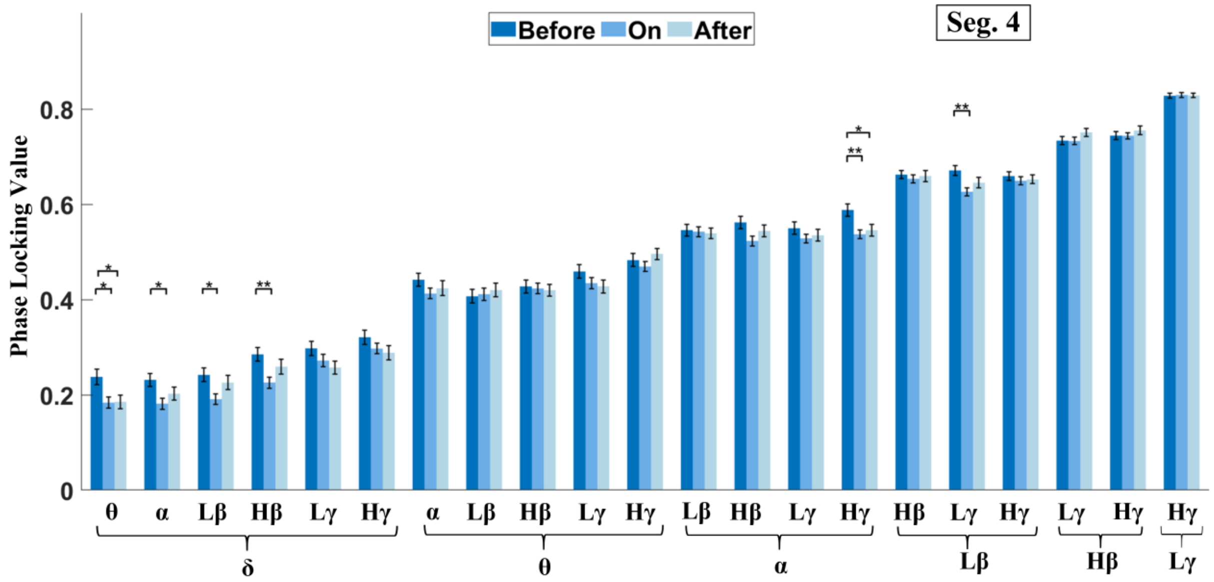

Supplementary Figure 1. Comparisons of PLVs in different pairs of frequency bands among the three different sound conditions, wherein each 2-s complete gait cycle was parsed into four 0.5-s step-phases, including (**A)** contralateral heel strike (Seg. 1), (**B**) contralateral foot stand (Seg. 2), (**C**) ipsilateral heel strike (Seg. 3), and (**D)** ipsilateral foot stand (Seg. 4).
